# Diagnostic utility of *RAS* mutation testing for refining cytologically indeterminate thyroid nodules

**DOI:** 10.17179/excli2024-6975

**Published:** 2024-02-15

**Authors:** Isabel R. Riccio, Alexandra C. LaForteza, Mohammad H. Hussein, Joshua P. Linhuber, Peter P. Issa, Jonathan Staav, Manal S. Fawzy, Eman A. Toraih, Emad Kandil

**Affiliations:** 1 School of Medicine, Tulane University, New Orleans, LA 70112, USA; 2 Division of Endocrine and Oncologic Surgery, Department of Surgery, School of Medicine, Tulane University, New Orleans, LA 70112, USA; 3 Department of Biochemistry, Faculty of Medicine, Northern Border University, Arar, Saudi Arabia; 4 Genetics Unit, Department of Histology and Cell Biology, Faculty of Medicine, Suez Canal University, Ismailia 41522, Egypt

**Keywords:** genomic mutations, indeterminate thyroid nodules, fine needle aspiration cytology, thyroid carcinoma, diagnostic accuracy, personalized medicine

## Abstract

*RAS* mutations are prevalent in indeterminate thyroid nodules, but their association with malignancy risk and utility for diagnosis remains unclear. We performed a systematic review and meta-analysis to establish the clinical value of *RAS* mutation testing for cytologically indeterminate thyroid nodules. PubMed and Embase were systematically searched for relevant studies. Thirty studies comprising 13,328 nodules met the inclusion criteria. Random effects meta-analysis synthesized pooled estimates of *RAS* mutation rates, risk of malignancy with *RAS* positivity, and histologic subtype outcomes. The pooled mutation rate was 31 % (95 % CI 19-44 %) among 5,307 indeterminate nodules. N*RAS* mutations predominated at 67 % compared to H*RAS* (24 %) and K*RAS* (12 %). The malignancy rate with *RAS* mutations was 58 % (95 %CI=48-68 %). *RAS* positivity increased malignancy risk 1.7-fold (RR 1.68, 95 %CI=1.21-2.34, p=0.002), with significant between-study heterogeneity (I2=89 %). Excluding one outlier study increased the relative risk to 1.75 (95 %CI=1.54-1.98) and I2 to 14 %. Funnel plot asymmetry and Egger's test (p=0.03) indicated potential publication bias. Among *RAS*-positive malignant nodules, 38.6 % were follicular variant papillary carcinoma, 34.1 % classical variant, and 23.2 % follicular carcinoma. No statistically significant difference in the odds of harboring *RAS* mutation was found between subtypes. In conclusion, *RAS* mutation testing demonstrates clinical utility for refining the diagnosis of cytologically indeterminate thyroid nodules. Positivity confers a 1.7-fold increased malignancy risk, supporting use for personalized decision-making regarding surgery vs. monitoring. Follicular variant papillary carcinoma constitutes the most common *RAS*-positive malignant histological subtype.

See also the graphical abstract(Fig. 1).

## Introduction

Thyroid nodules are commonly encountered in clinical practice and are detected in up to 65 % of the general population (Dean and Gharib, 2008[15]). At the same time, most nodules are benign, and approximately 10-15 % harbor malignancy (Kamran et al., 2013[29]). Fine needle aspiration (FNA) cytology is the standard diagnostic test for thyroid nodules, and the “Bethesda System for Reporting Thyroid Cytopathology” is a widely accepted framework that classifies thyroid FNA biopsy results into six diagnostic categories, with each category reflecting a distinct likelihood of malignancy (Haugen et al., 2016[27]). In brief, the categories include I) non-diagnostic or unsatisfactory, II) benign, III) atypia of undetermined significance or follicular lesion of undetermined significance (AUS/FLUS), IV) follicular neoplasm or suspicious for a follicular neoplasm, V) suspicious for malignancy, and VI) malignant. Categories III, IV, and V are considered indeterminate and pose challenges in clinical management because they do not clearly distinguish between benign and malignant lesions. Consequently, these categories often require further diagnostic procedures, such as molecular testing, to guide clinical decisions (Haugen et al., 2016[27]).

The risk of malignancy for Bethesda III nodules ranges from 6-48 %, while Bethesda IV carries a 16-73 % risk, and Bethesda V has a 60-75 % risk (Singh and Wang, 2011[45]). Due to this uncertainty, most patients with indeterminate nodules are referred for diagnostic thyroid surgery; however, only 20-37 % prove to be malignant on final pathology (Yang et al., 2007[54]). This leads to potential overtreatment and unnecessary surgical risks.

Various molecular markers have been studied to improve the preoperative diagnosis of indeterminate nodules, including *BRAF *and *RAS* point mutations, *RET/PTC* rearrangements, and galectin-3 immunostaining (Bartolazzi et al., 2018[4]; Ferrari et al., 2018[20]; Lu et al., 2023[32]; Patel et al., 2017[40]). Studies have demonstrated up to 70 % of papillary thyroid cancer harbors a mutation (Oishi et al., 2017[39], Prete et al., 2020[41]). Among these, *RAS* mutations (in *NRAS*, *HRAS*, and *KRAS* genes) are found in 10-20 % of papillary thyroid cancers (Gilani et al., 2022[21]). Several studies have evaluated *RAS* testing specifically in nodules with indeterminate cytology, with mutation rates of 8.5 %-72 % reported (An et al., 2015[1]; Macerola et al., 2019[34]; Marotta et al., 2021[35]). 

While several individual studies have been published, the diagnostic utility and clinical significance of *RAS* mutations in indeterminate nodules remain unclear. In this sense, we performed a systematic review and meta-analysis to synthesize the existing evidence on 1) the frequency of *RAS* mutations in indeterminate thyroid nodules and 2) the association between *RAS* mutation status and risk of malignancy. Additionally, we examined the relationship between *RAS* mutations and histologic subtypes of malignancy. Our findings aim to clarify the diagnostic utility of *RAS* mutation testing for triaging indeterminate nodules.

## Materials and Methods

### Search strategy

This meta-analysis followed the Preferred Reporting Items for Systematic Review and Meta-Analyses (PRISMA) guidelines. We systematically searched PubMed and Embase to cover the published articles between 2010-2024, using a combination of relevant keywords related to indeterminate thyroid nodules, fine needle aspiration, *RAS* mutations, and thyroid cancer. We manually screened the reference lists of included studies and relevant reviews for additional eligible studies. 

### Selection criteria

Studies were included if they met the following criteria: (1) evaluated indeterminate thyroid nodules (Bethesda category III, IV, or V) diagnosed by FNA, (2) assessed *RAS* mutational status, (3) had surgical histopathological confirmation of nodule diagnosis, (4) reported data to calculate the proportion of *RAS* mutations and/or the association between *RAS* status and cancer risk. Case reports, conference abstracts, editorials, and non-English studies were excluded.

Three investigators independently screened the titles, abstracts, and full texts of retrieved studies against the eligibility criteria. Disagreements were resolved by consensus or consultation with a fourth investigator if needed.

### Data extraction 

Three investigators used a standardized form to extract data from the included studies on study characteristics (author, year, country, design), patient characteristics (age, sex, nodule size), FNA details (Bethesda category, sample size), number of nodules tested for *RAS*, number of *RAS* mutations, final surgical pathology diagnosis, and data for 2x2 tables of *RAS* status (positive/negative) versus malignancy (positive/negative).

### Study outcomes

The primary outcomes were the pooled proportion of RAS mutations in indeterminate nodules and the relative risk (RR) of malignancy associated with RAS mutation positivity. Secondary outcomes included the odds ratio for the histological subtype of malignancy (classical/follicular variant papillary thyroid cancer).

### Single proportion meta-analysis

We calculated the pooled proportion of 1) Bethesda category III/IV/V nodules and 2) the rate of malignancy among surgically resected indeterminate nodules using the Freeman-Tukey double arcsine transformation to stabilize the variances and then summarized the proportions using a random effects meta-analysis model. For the rate of RAS mutations in indeterminate nodules, we used an arcsine square-root transformation to account for studies with RAS mutation rates of zero. We pooled the transformed proportions using a random effects model.

### Pairwise comparison meta-analysis

We used a Mantel-Haenszel random effects model to estimate the relative risk of malignancy associated with RAS mutation positivity (Dettori et al., 2022[17]). The between-study variance was estimated using the DerSimonian-Laird method (Bakbergenuly et al., 2020[2]). We added a continuity correction of 0.5 to studies with zero events.

For the secondary outcome of the histological subtype, we used a Mantel-Haenszel random effects model to calculate the odds ratio of classical variant/follicular variant papillary carcinoma associated with RAS mutations. We used the same methods as above to quantify and test for heterogeneity.

### Heterogeneity analysis

For all proportion meta-analyses, we quantified heterogeneity using the I^2^ statistic and tested for heterogeneity using Cochran's Q test (West et al., 2010[52]). We used the Knapp-Hartung modification to account for uncertainty in the estimated variance of each study (Jackson et al., 2017[28]). Confidence intervals for individual studies were calculated using the Clopper-Pearson method (Jackson et al., 2017[28]).

### Evaluation of publication bias

We assessed publication bias through visual inspection of funnel plots and quantitatively using Egger's regression test (Egger et al., 1997[18]). For estimates with evidence of publication bias, we performed trim-and-fill analysis to adjust for hypothetical missing studies.

### Sensitivity analyses and meta-regression analysis

We conducted sensitivity analyses excluding studies with a high risk of bias, studies with inadequate sample size, and other subgroup analyses to evaluate the robustness of findings. All analyses were done in R version 4.0.2 using the *meta *R and *metafor *packages.

## Results

### Literature search

**Figure 2:**
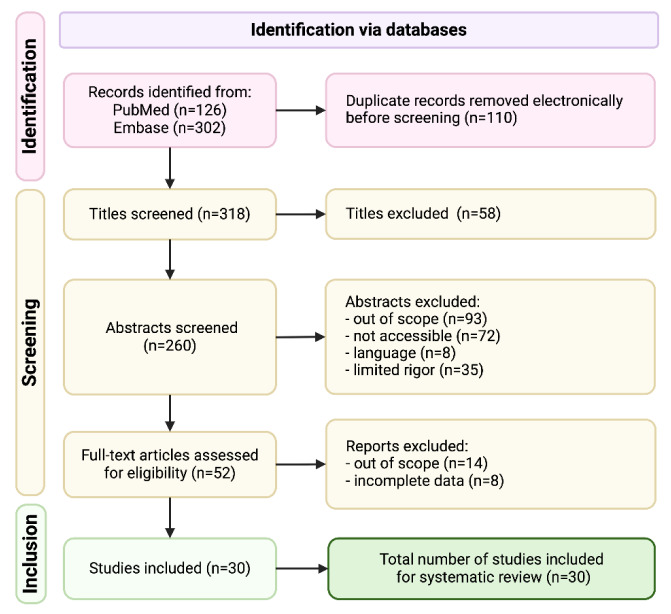
Study selection by PRISMA flow diagram

A systematic literature search identified 318 potentially relevant studies. After screening titles and abstracts, 52 articles were retrieved for full-text review. Thirty studies with 13,328 nodules in 12,338 patients met the final inclusion criteria (Figure 2(Fig. 2)).

### Study characteristics

The 30 included studies comprised 16 prospective and 14 retrospective designs, conducted across 10 countries from 2010 to 2022, including 10 studies from the USA (Beaudenon-Huibregtse et al., 2014[5]; Belovarac et al., 2022[6]; Guan et al., 2020[24]; Gupta et al., 2013[25]; Lupo et al., 2020[33]; Moses et al., 2010[37]; Nikiforov et al., 2011[38]; Shrestha et al., 2016[44]; Stence et al., 2015[47]; Valderrabano et al., 2017[50]), five from Italy (Cantara et al., 2010[8]; Censi et al., 2017[9]; Colombo et al., 2021[13]; De Napoli et al., 2016[14]; Macerola et al., 2019[34]), and four from China (Liu et al., 2014[30]; Lu et al., 2021[31]; Song et al., 2020[46]; Wu et al., 2019[53]), among others (An et al., 2015[1]; Bardet et al., 2015[3]; Chen et al., 2020[10]; Cho et al., 2020[11]; Decaussin-Petrucci et al., 2017[16]; Eszlinger et al., 2014[19]; Gill et al., 2015[22]; Grimmichova et al., 2022[23]; Ravella et al., 2020[43]; Tolaba et al., 2021[49]; Vishwanath et al., 2022[51]). Sample sizes ranged from 42 to 2,306 nodules evaluated by FNA. About 5,307 nodules were diagnosed as indeterminate, of which 3,327 underwent surgical resection with histopathological confirmation (Table 1(Tab. 1); References in Table 1: An, 2015[1]; Bardet, 2015[3]; Beaudenon-Huibregtse, 2014[5]; Belovarac, 2022[6]; Cantara, 2010[8]; Censi, 2017[9]; Chen, 2020[10]; Cho, 2020[11]; Colombo, 2021[13]; De Napoli, 2016[14]; Decaussin-Petrucci, 2017[16]; Eszlinger, 2014[19]; Gill, 2015[22]; Grimmichova, 2022[23]; Guan, 2020[24]; Gupta, 2013[25]; Liu, 2014[30]; Lu, 2021[31]; Lupo, 2020[33]; Macerola, 2019[34]; Moses, 2010[37]; Nikiforov, 2011[38]; Ravella, 2020[43]; Shrestha, 2016[44]; Song, 2020[46]; Stence, 2015[47]; Tolaba, 2021[49]; Valderrabano, 2017[50]; Vishwanath, 2022[51]; Wu, 2019[53]).

### Pooled rates in indeterminate nodules

**Figure 3:**
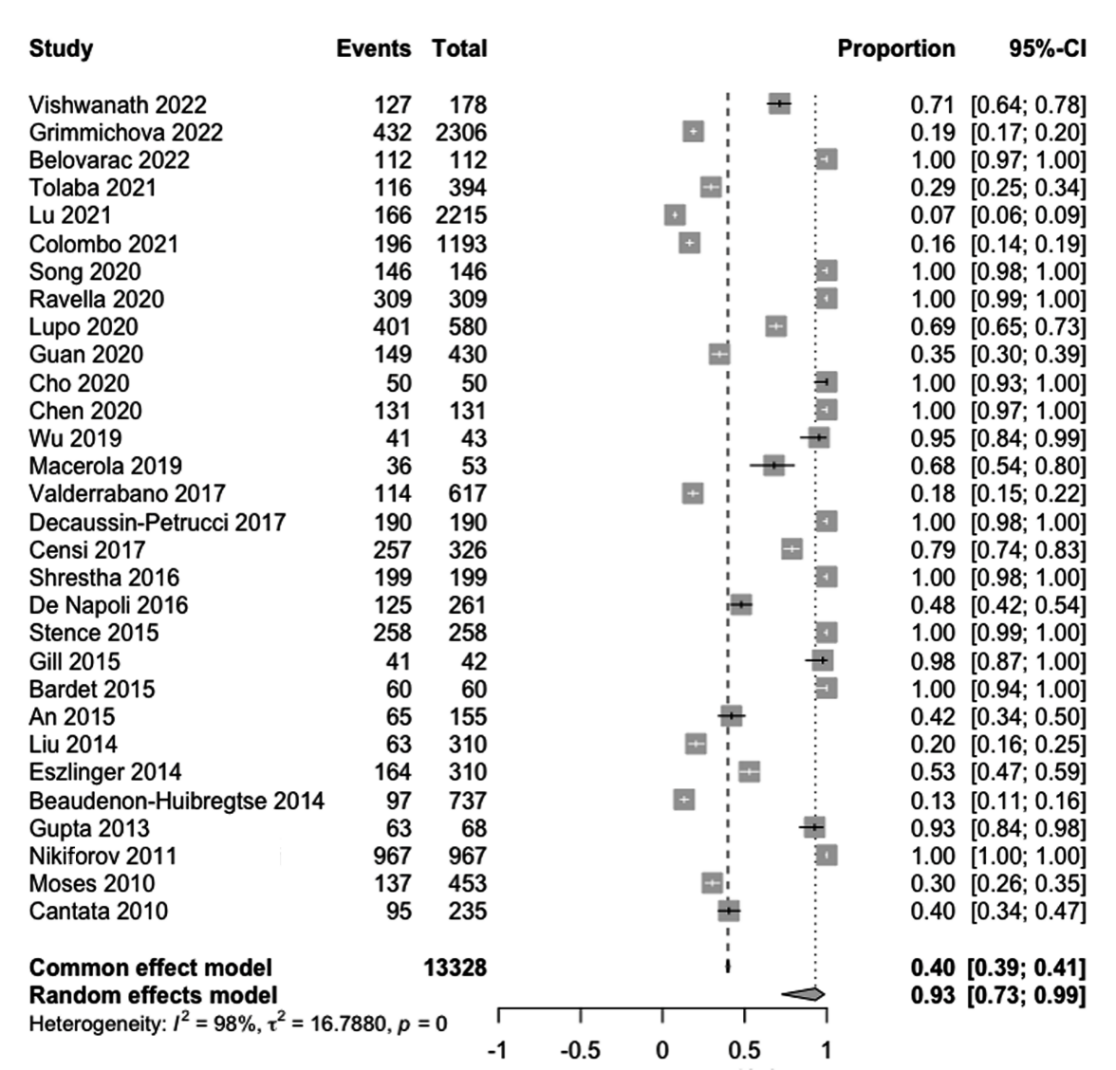
Forest plots show a pooled proportion of indeterminate thyroid nodules across the studies

The pooled proportion of Bethesda category III/IV/V nodules was 93 % (95 % CI, 73-99 %) (Figure 3(Fig. 3); References in Figure 3: An, 2015[1]; Bardet, 2015[3]; Beaudenon-Huibregtse, 2014[5]; Belovarac, 2022[6]; Cantara, 2010[8]; Censi, 2017[9]; Chen, 2020[10]; Cho, 2020[11]; Colombo, 2021[13]; De Napoli, 2016[14]; Decaussin-Petrucci, 2017[16]; Eszlinger, 2014[19]; Gill, 2015[22]; Grimmichova, 2022[23]; Guan, 2020[24]; Gupta, 2013[25]; Liu, 2014[30]; Lu, 2021[31]; Lupo, 2020[33]; Macerola, 2019[34]; Moses, 2010[37]; Nikiforov, 2011[38]; Ravella, 2020[43]; Shrestha, 2016[44]; Song, 2020[46]; Stence, 2015[47]; Tolaba, 2021[49]; Valderrabano, 2017[50]; Vishwanath, 2022[51]; Wu, 2019[53]). The frequency ranged from 13 % to 100 %.

**Figure 4:**
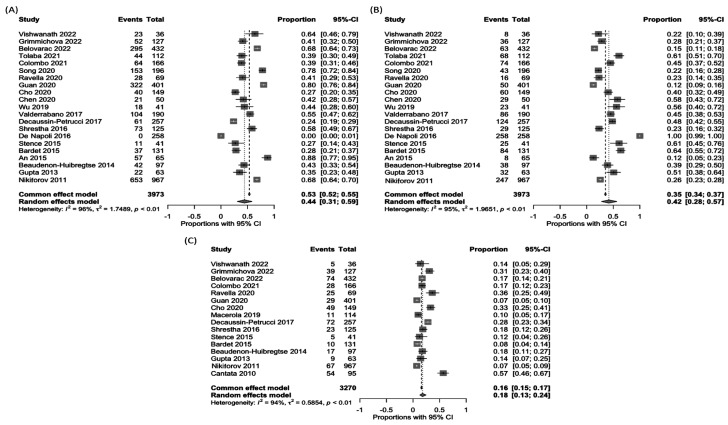
Forest plots show the pooled proportion of Bethesda III (A), IV (B), and V (C) subtype nodules

For indeterminate subtypes, atypia of undetermined significance or follicular lesion of undetermined significance (AUS/FLUS) occurred in 44 % (95 % CI, 31-59 %), follicular neoplasm (FN) in 42 % (95 % CI, 28-57 %), and suspicious for malignancy (SUSP) in 18 % (95 % CI, 13-24 %) (Figure 4(Fig. 4); References in Figure 4: An, 2015[1]; Bardet, 2015[3]; Beaudenon-Huibregtse, 2014[5]; Belovarac, 2022[6]; Cantara, 2010[8]; Censi, 2017[9]; Chen, 2020[10]; Cho, 2020[11]; Colombo, 2021[13]; De Napoli, 2016[14]; Decaussin-Petrucci, 2017[16]; Eszlinger, 2014[19]; Gill, 2015[22]; Grimmichova, 2022[23]; Guan, 2020[24]; Gupta, 2013[25]; Liu, 2014[30]; Lu, 2021[31]; Lupo, 2020[33]; Macerola, 2019[34]; Moses, 2010[37]; Nikiforov, 2011[38]; Ravella, 2020[43]; Shrestha, 2016[44]; Song, 2020[46]; Stence, 2015[47]; Tolaba, 2021[49]; Valderrabano, 2017[50]; Vishwanath, 2022[51]; Wu, 2019[53]).

### Mutation rate of RAS genes in indeterminate nodules

**Figure 5:**
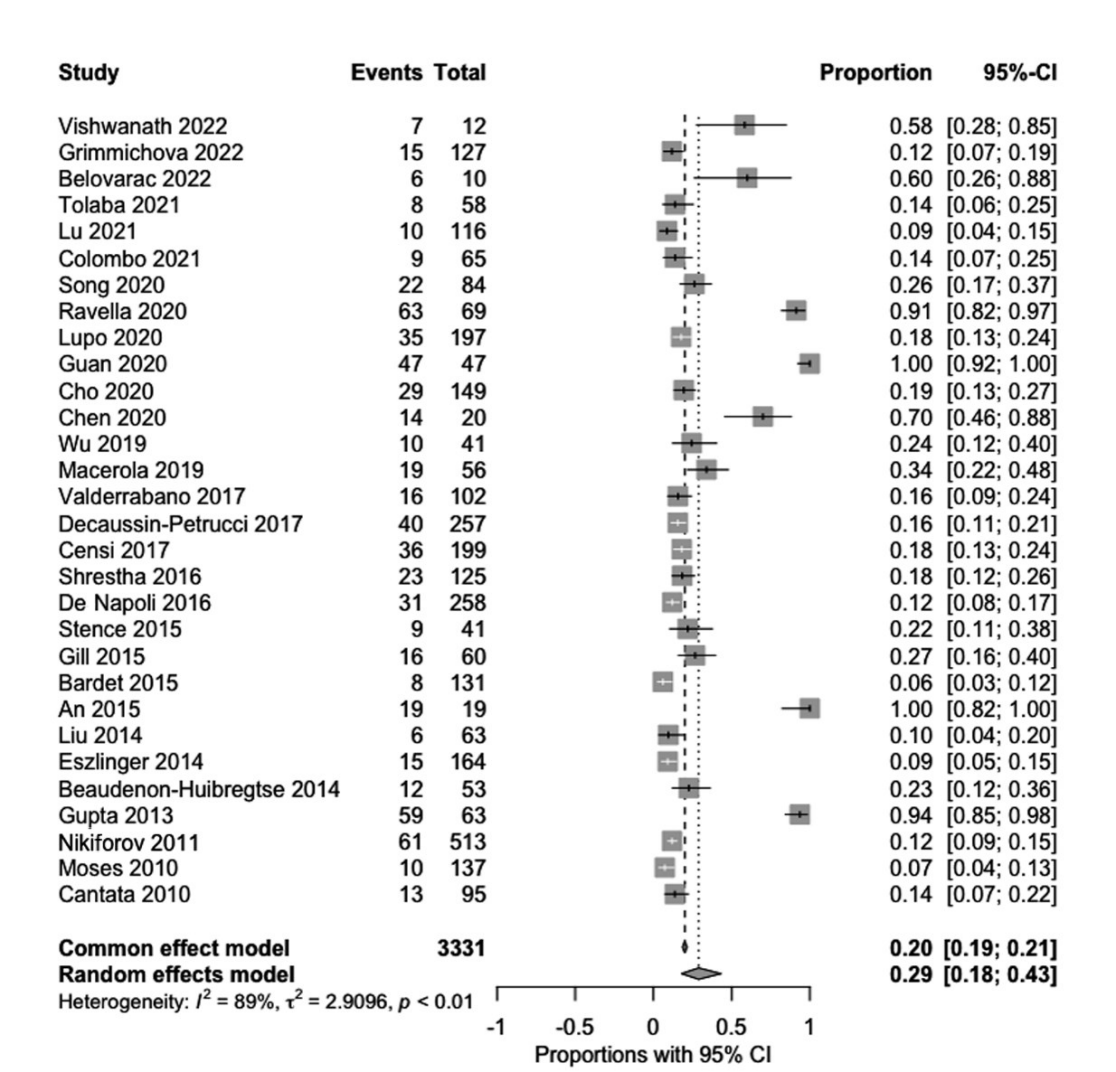
Rate of *RAS* mutation in surgically resected indeterminate nodules

Out of the whole population, 91 % (95 % CI, 76 %-97 %) of patients with indeterminate nodules underwent surgery. Of these, 29 % (95 % CI, 18 %-43 %) had positive *RAS* mutation (Figure 5(Fig. 5); References in Figure 5: An, 2015[1]; Bardet, 2015[3]; Beaudenon-Huibregtse, 2014[5]; Belovarac, 2022[6]; Cantara, 2010[8]; Censi, 2017[9]; Chen, 2020[10]; Cho, 2020[11]; Colombo, 2021[13]; De Napoli, 2016[14]; Decaussin-Petrucci, 2017[16]; Eszlinger, 2014[19]; Gill, 2015[22]; Grimmichova, 2022[23]; Guan, 2020[24]; Gupta, 2013[25]; Liu, 2014[30]; Lu, 2021[31]; Lupo, 2020[33]; Macerola, 2019[34]; Moses, 2010[37]; Nikiforov, 2011[38]; Ravella, 2020[43]; Shrestha, 2016[44]; Song, 2020[46]; Stence, 2015[47]; Tolaba, 2021[49]; Valderrabano, 2017[50]; Vishwanath, 2022[51]; Wu, 2019[53]). Among *RAS* mutations, N*RAS* mutations predominated at 67 % (95 % CI, 55-79 %), followed by H*RAS* 24 % (95 % CI, 19-29 %) and K*RAS* 12 % (95 % CI, 5-22 %).

### Rates of malignancy in RAS positive indeterminate nodules

**Figure 6:**
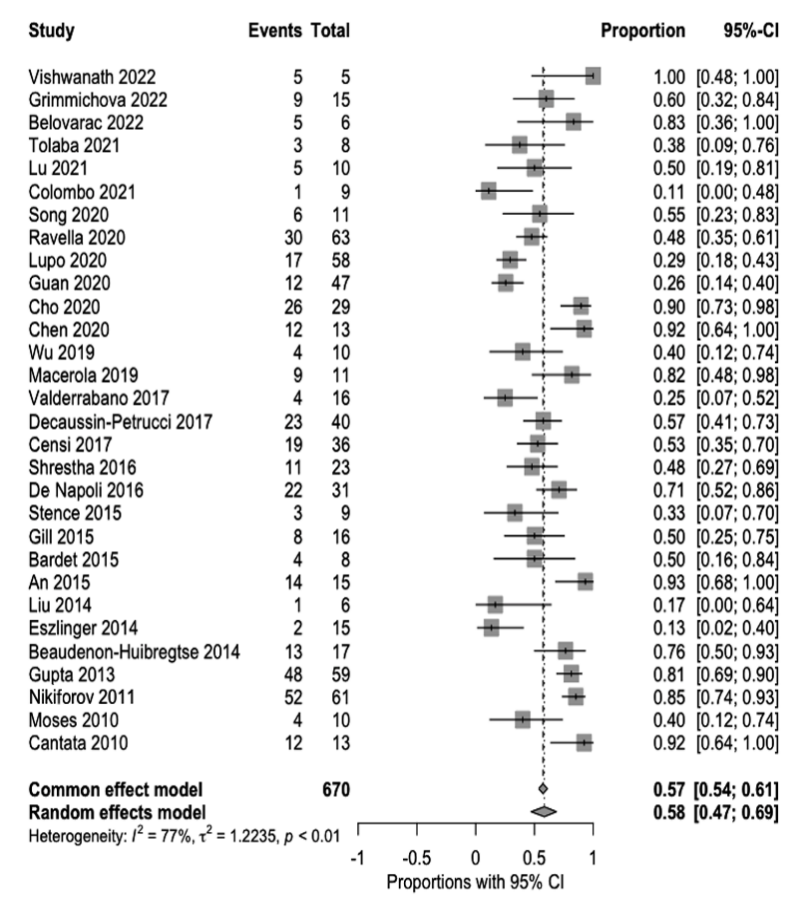
Figure 6: Rate of malignancy in *RAS*-positive surgically resected indeterminate thyroid nodules

In surgically resected indeterminate nodules, 31 % (95 % CI 19-44 %) harbored malignancy. Specifically, in indeterminate nodules with positive *RAS* mutation, the malignancy rate was 58 % (95 % CI, 47 %-69 %) (Figure 6(Fig. 6); References in Figure 6: An, 2015[1]; Bardet, 2015[3]; Beaudenon-Huibregtse, 2014[5]; Belovarac, 2022[6]; Cantara, 2010[8]; Censi, 2017[9]; Chen, 2020[10]; Cho, 2020[11]; Colombo, 2021[13]; De Napoli, 2016[14]; Decaussin-Petrucci, 2017[16]; Eszlinger, 2014[19]; Gill, 2015[22]; Grimmichova, 2022[23]; Guan, 2020[24]; Gupta, 2013[25]; Liu, 2014[30]; Lu, 2021[31]; Lupo, 2020[33]; Macerola, 2019[34]; Moses, 2010[37]; Nikiforov, 2011[38]; Ravella, 2020[43]; Shrestha, 2016[44]; Song, 2020[46]; Stence, 2015[47]; Tolaba, 2021[49]; Valderrabano, 2017[50]; Vishwanath, 2022[51]; Wu, 2019[53]).

### Risk of malignancy 

**Figure 7:**
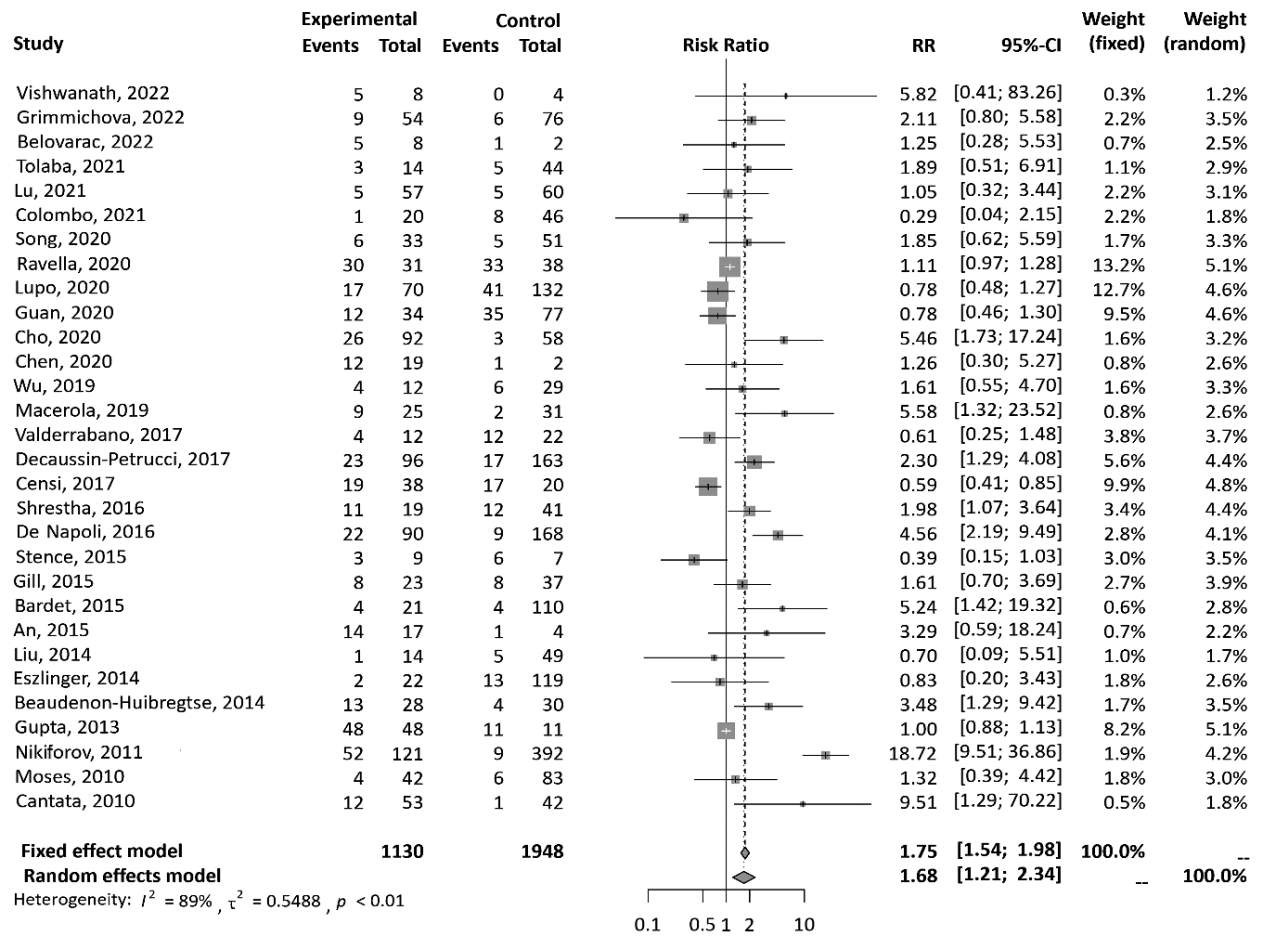
Forest plots show the relative risk of malignancy conferred by *RAS* mutation positivity

*RAS* mutation positivity conferred a 1.68-fold higher risk of malignancy (RR 1.68, 95 % CI, 1.21-2.34, *p*=0.002) (Figure 7(Fig. 7); References in Figure 7: An, 2015[1]; Bardet, 2015[3]; Beaudenon-Huibregtse, 2014[5]; Belovarac, 2022[6]; Cantara, 2010[8]; Censi, 2017[9]; Chen, 2020[10]; Cho, 2020[11]; Colombo, 2021[13]; De Napoli, 2016[14]; Decaussin-Petrucci, 2017[16]; Eszlinger, 2014[19]; Gill, 2015[22]; Grimmichova, 2022[23]; Guan, 2020[24]; Gupta, 2013[25]; Liu, 2014[30]; Lu, 2021[31]; Lupo, 2020[33]; Macerola, 2019[34]; Moses, 2010[37]; Nikiforov, 2011[38]; Ravella, 2020[43]; Shrestha, 2016[44]; Song, 2020[46]; Stence, 2015[47]; Tolaba, 2021[49]; Valderrabano, 2017[50]; Vishwanath, 2022[51]; Wu, 2019[53]).

### Evaluation of bias and heterogeneity analysis

**Figure 8:**
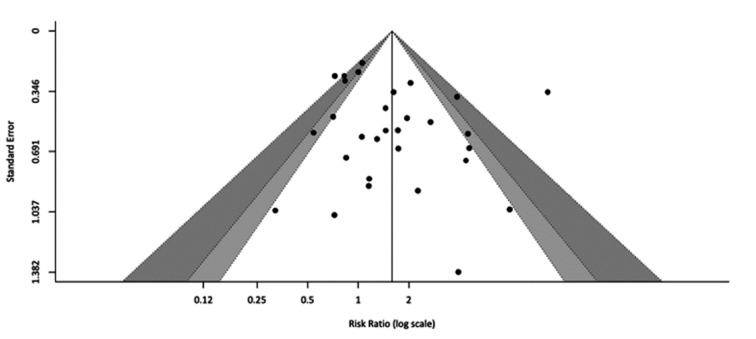
Funnel plot assessing publication bias in the relative risk analysis

**Figure 9:**
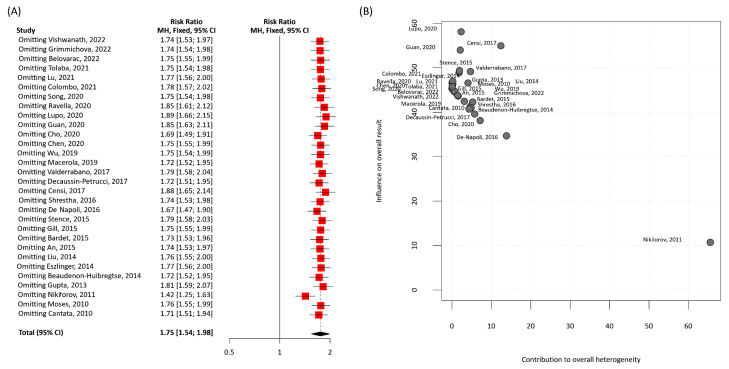
Heterogeneity analysis

There was evidence of publication bias by Egger's test (*p*=0.03) and funnel plot asymmetry (Figure 8(Fig. 8)). Sensitivity analysis and Baujat plot indicated that heterogeneity was partly explained by one outlier study (Nikiforov et al., 2011[38]). This study was removed in a revised analysis for the relative risk of malignancy in the presence of *RAS* mutation (RR:1.75, 95 % CI 1.54-1.98) (Figure 9(Fig. 9); References in Figure 9: An, 2015[1]; Bardet, 2015[3]; Beaudenon-Huibregtse, 2014[5]; Belovarac, 2022[6]; Cantara, 2010[8]; Censi, 2017[9]; Chen, 2020[10]; Cho, 2020[11]; Colombo, 2021[13]; De Napoli, 2016[14]; Decaussin-Petrucci, 2017[16]; Eszlinger, 2014[19]; Gill, 2015[22]; Grimmichova, 2022[23]; Guan, 2020[24]; Gupta, 2013[25]; Liu, 2014[30]; Lu, 2021[31]; Lupo, 2020[33]; Macerola, 2019[34]; Moses, 2010[37]; Nikiforov, 2011[38]; Ravella, 2020[43]; Shrestha, 2016[44]; Song, 2020[46]; Stence, 2015[47]; Tolaba, 2021[49]; Valderrabano, 2017[50]; Vishwanath, 2022[51]; Wu, 2019[53]).

### Histopathological subtypes

Among RAS-positive malignant thyroid nodules, the meta-analysis found that the follicular variant of papillary thyroid cancer (FV-PTC) constituted 38.60 % (95 % CI 23.40-56.30 %) of cases. The classical variant of papillary thyroid cancer (CV-PTC) represented 34.10 % (95 % CI 19.50-52.40 %) of cases. On the other hand, follicular thyroid cancer (FTC) accounted for 23.20 % (95 % CI 14.70-34.50 %) of RAS-positive malignant nodules. Significant heterogeneity was detected across the pooled estimates (I2>50 %) (Table 2(Tab. 2)).

Furthermore, pairwise comparisons between the subtypes revealed no statistically significant differences in the odds of harboring *RAS* mutations. Specifically, the odds ratio for FV-PTC versus CV-PTC was 1.06 (95 % CI 0.25-4.37; *p*=0.93). The FTC versus CV-PTC comparison yielded an odds ratio of 0.64 (95 % CI 0.22-1.82; *p*=0.40), and the FTC versus FV-PTC comparison showed an odds ratio of 0.51 (95 % CI 0.15-1.68; *p*=0.26) (Table 3(Tab. 3)).

## Discussion

In this systematic review and meta-analysis of 30 studies with over 13,000 thyroid nodules, we found that *RAS* mutations are prevalent in approximately 30 % of nodules with indeterminate fine needle aspiration cytology. A *RAS* mutation was associated with a 1.7-fold increased risk of malignancy. Among *RAS*-positive malignant nodules, the odds of classical variant papillary thyroid carcinoma were nearly ten times higher compared to follicular carcinoma. These findings suggest that *RAS* mutational testing may have clinical utility for refining the diagnosis of indeterminate nodules.

The 29 % pooled rate of *RAS* mutations we identified is within the 18-43 % generally reported for sporadic papillary thyroid cancers (Zhou et al., 2023[55]). However, prior studies in indeterminate nodules have reported *RAS* mutation frequencies of 26-34 %, more concordant with our estimate (Gill et al., 2015[22]; Macerola et al., 2019[34]; Song et al., 2020[46]). The prevalence likely reflects enrichment for *RAS* mutations among cytologically indeterminate nodules.

We found *RAS* positivity significantly predicts malignancy, conferring a 1.7-fold increased risk, although the data exhibited heterogeneity. This aligns with recent studies demonstrating that molecular profiling adds incremental diagnostic value over clinical features and ultrasound for thyroid nodules with indeterminate cytology (Morand et al., 2024[36]; Stewardson et al., 2023[48]). Specifically, a previous meta-analysis found that *RAS*-mutated indeterminate nodules have a positive predictive value of 78 % and a positive likelihood ratio of 4.23, suggesting further investigation into the *RAS* mutation in indeterminate nodules for diagnostic accuracy (Clinkscales et al., 2017[12]).

A recent multicenter, multinational retrospective study examined thyroid nodules with a wider range of Bethesda III to VI cytology through expansive molecular profiling, including ThyGenX/ThyGeNEXT and ThyroSeq V3 tests (Morand et al., 2024[36]). Notably, the study identified RAS-like alterations predominantly in Bethesda III and IV nodules, which aligns with the focus of our meta-analysis. These RAS-like mutations were associated with a lower likelihood of extrathyroidal extension, nodal disease, and aggressive histology, which further illuminates the diagnostic subtleties that RAS mutation testing can bring to the clinical evaluation of indeterminate thyroid nodules. However, while this study provides valuable insights into the potential for molecular testing to predict malignant behavior and guide patient management, it encompasses a broader range of nodules than our analysis, including Bethesda VI and a variety of molecular alterations beyond those in the RAS gene family. The inclusion of a mix of RAS-like and non-RAS mutations, and especially the inclusion of Bethesda VI category nodules, which are known to have a high likelihood of malignancy, suggest divergent clinical implications that are beyond the scope of our investigation, thus not meeting our eligibility criteria. Nevertheless, their findings complement our results by highlighting the importance and utility of RAS mutation testing among the array of molecular diagnostic tools. Future studies could build upon these results, examining the implications of conducting molecular profiling that targets specific mutation types across a tightly defined range of Bethesda categories, ultimately contributing to a more personalized approach to thyroid nodule management.

It is worth noting that the risk conferred by *RAS* mutations appears more modest than other markers like *BRAF*^V600E^, which carry higher specificity for papillary thyroid cancer (Zou et al., 2014[56]).

Our study estimates the distribution of *RAS* mutation subtypes in indeterminate nodules, with N*RAS* codominant at 67 %, followed by H*RAS* and K*RAS*. Previous reviews have suggested N*RAS* mutations predominate but lacked sufficient data to quantify the relative distribution (Clinkscales et al., 2017[12]). *RAS* subtype may have implications for prognostication, as N*RAS* and K*RAS* mutations have been associated with less aggressive disease, whereas H*RAS* mutations confer a higher probability of carcinoma outcome risk (Radkay et al., 2014[42]).

Regarding histopathological outcomes, our meta-analysis found *RAS*-positive malignant nodules harbored a distribution of 34 % classical variant PTC, 39 % follicular variant PTC, and 23 % follicular thyroid carcinoma. There was significant heterogeneity across studies for all subtype estimates. The predominance of classic and follicular variant PTC histologies likely reflects the known association between *RAS* mutations and papillary carcinoma, rather than follicular carcinoma, in thyroid malignant transformation (Cameselle-Teijeiro et al., 2020[7]). The lack of distinction in *RAS* mutation prevalence between subtypes indicates that cytological phenotype does not necessarily confer genotype specificity. Overall, *RAS* analysis can improve diagnostic accuracy without necessarily providing definitive subclassification capacity between PTC variants. Additional large-scale studies are still needed to clarify if subtle inter-subtype differences exist in the likelihood of harboring *RAS* mutations. 

Currently, the ATA guidelines issue a weak recommendation for molecular testing to help guide the management of cytologically indeterminate thyroid nodules (Haugen, 2017[26]). Based on our findings, we suggest that *RAS* analysis is a useful diagnostic adjunct for nodules with indeterminate cytology. Testing can be readily performed on FNA cytology samples prior to surgical decision-making. Given that, nearly half of the indeterminate nodules harbor *RAS* mutations, which confer an increased risk of malignancy, a positive *RAS* test result may prompt consideration of surgical excision or more frequent ult*RAS*ound follow-up instead of continued observation. Negative *RAS* testing suggests lower malignancy risk, providing reassurance for conservative management.

Incorporating *RAS* analysis into indeterminate thyroid nodule evaluation may reduce unnecessary surgeries for benign nodules and allow earlier diagnosis of *RAS*-positive classical variant papillary cancers. There is potential value for *RAS* subtyping as well. Additional studies are still needed to determine if *RAS* mutations can stratify malignant risk within the AUS/FLUS, FN, and SUSP Bethesda categories. Future research should also examine the prognostic significance and predictive value of *RAS* mutations regarding response to adjuvant therapy in thyroid cancer patients.

Several limitations should be considered when interpreting our meta-analysis. There was significant between-study heterogeneity, which may reflect differences in geographic cohorts, molecular methods, and histopathological classification across studies. To mitigate this issue, we conducted sensitivity analyses, which achieved homogeneity after excluding an outlier study. Additionally, eligible studies were observational. Consequently, the potential for residual confounding factors cannot be ruled out.

Furthermore, while focusing on *RAS* status, we recognize that other germline and somatic mutations might coexist and influence the outcomes, but these factors were not accounted for in our analysis due to data limitations. A significant gap in our meta-analysis is the inability to assess the prognostic significance of *RAS* mutations, mainly because most of the included studies did not provide follow-up data, which limits our understanding of the long-term implications of these mutations in the studied population. Finally, providing a nuanced understanding of how *RAS* mutation status might correlate with malignancy risk across different genders could have valuable implications for personalizing the management of thyroid nodules and is an essential suggestion for progressing the field.

## Conclusions

Our analysis highlights that while *RAS* testing can improve diagnostic accuracy, it does not necessarily offer definitive subclassification capacity between thyroid carcinoma variants. Given that a significant proportion of indeterminate nodules harbor *RAS* mutations with an associated increased malignancy risk, a positive *RAS* test may influence management decisions, from surgical excision to more intensive monitoring, whereas negative results may support conservative management. In conclusion, incorporating *RAS* analysis in evaluating indeterminate thyroid nodules could reduce unnecessary surgeries and facilitate earlier diagnosis of certain cancers. Further studies are warranted to determine the prognostic significance of *RAS* mutations.

## Notes

Manal S. Fawzy and Eman A. Toraih (Division of Endocrine and Oncologic Surgery, Department of Surgery, School of Medicine, Tulane University, New Orleans, LA 70112, USA; E-mail: etoraih@tulane.edu) contributed equally as corresponding author.

## Declaration

### Author contributions

Conceptualization: EAT, EK; Data curation: IRR, ACL, JPL, PPI, JS; Formal analysis: MHH, EAT; Funding acquisition: EAT; Methodology: IRR, ACL, JPL, PPI, JS, EAT; Software: MHH, EAT; Supervision: EAT, EK; Validation: MHH, MSF, EK; Writing - original draft: MHH, MSF, EAT; Writing - review & editing: IRR, ACL, MHH, JPL, PPI, JS, MSF, EAT, EK. All authors have read and agreed to the published version of the manuscript.

### Funding

The project described was supported by ThyCa: Thyroid Cancer Survivors' Association, Inc. and administered by the American Thyroid Association through grant number [THYROIDGRANT2021-0000000232] and The School of Medicine Pilot Grant (to ET). Furthermore, the Deanship of Scientific Research at Northern Border University, Arar, KSA, funded this research work through the project number "NBU-FFR-2024-1442-02" (to MSF).

### Conflict of interest

The authors declare no conflict of interest.

## Figures and Tables

**Table 1:**
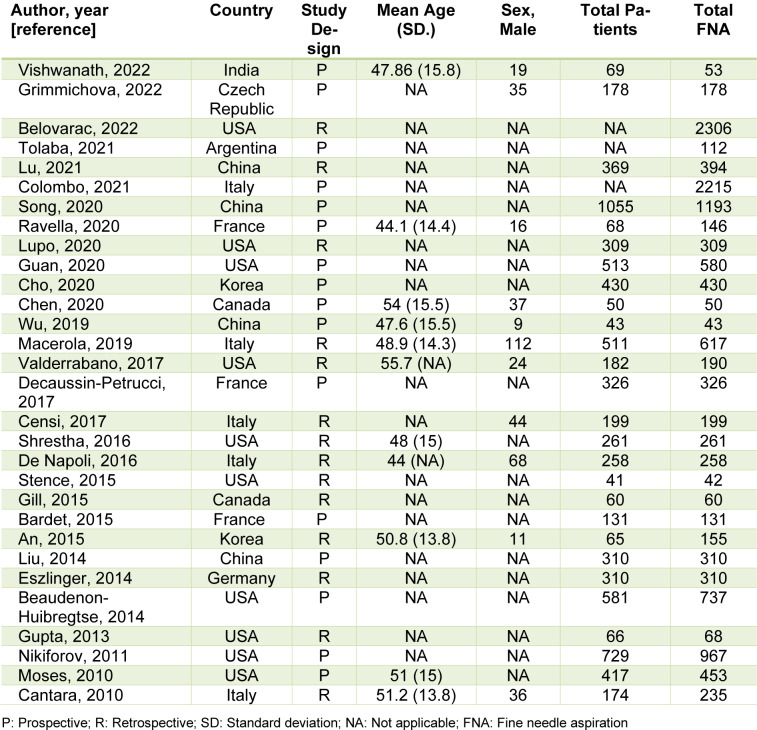
Eligible article characteristics

**Table 2:**
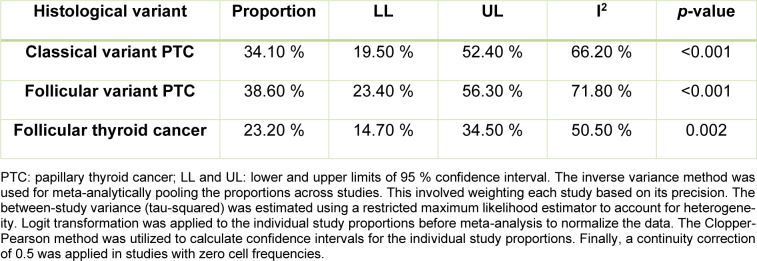
Pooled proportion of pathological variant among *RAS*-positive malignant nodules

**Table 3:**
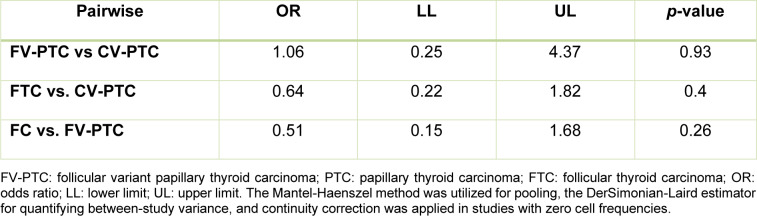
Pairwise subtype comparison based on histopathological types

**Figure 1:**
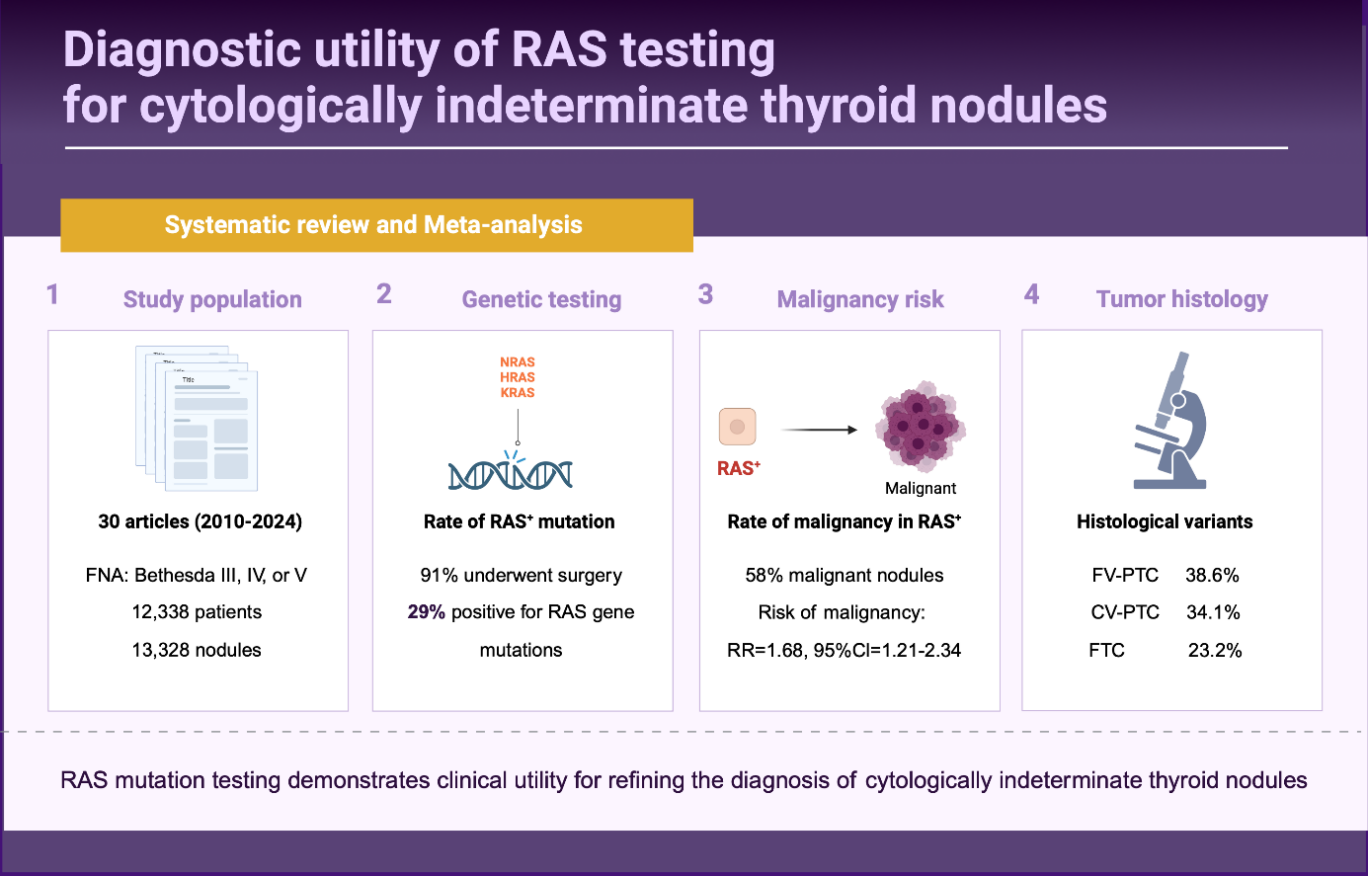
Graphical abstract
